# The potential role of telemedicine in the infectious disease pandemic with an emphasis on COVID‐19: A narrative review

**DOI:** 10.1002/hsr2.1024

**Published:** 2023-01-03

**Authors:** Fazlollah Shokri, Sara Bahrainian, Fatemeh Tajik, Elaheh Rezvani, Aref Shariati, Shima nourigheimasi, Elahe Saberi Shahrebabaki, Maryam Ebrahimi, Farhan Shamoon, Mohsen Heidary

**Affiliations:** ^1^ Department of Medical Genetics Faculty of Medicine, Shahid Beheshti University of Medical Sciences Tehran Iran; ^2^ Department of Food and Drug Control School of Pharmacy, Ahvaz Jundishapur University of Medical Sciences Ahvaz Iran; ^3^ Faculty of Medicine, Iran University of Medical Sciences Tehran Iran; ^4^ Department of Genetics, Faculty of Advanced Science and Technology, Tehran Medical Sciences Islamic Azad University Tehran Iran; ^5^ Molecular and medicine research center, Khomein University of Medical Sciences Khomein Iran; ^6^ Faculty of Medicine, Arak University of Medical Sciences Arak Iran; ^7^ Tracheal Diseases Research Center, National Research Institute of Tuberculosis and Lung Diseases Shahid Beheshti University of Medical Sciences Tehran Iran; ^8^ Faculty of Pharmacy, Tehran University of Medical Sciences Tehran Iran; ^9^ Student Research Committee, Sabzevar University of Medical Sciences Sabzevar Iran; ^10^ Cellular and Molecular Research Center, Sabzevar University of Medical Sciences Sabzevar Iran

**Keywords:** COVID‐19, health systems, infectious disease pandemic, telemedicine

## Abstract

**Background and Aims:**

Due of its low cost, rapid speed, data record, and vast communication coverage, information and communication technology might be useful for health‐related fields in times of crisis. By providing medical or hygienic services to a patient who lives elsewhere using communication methods like email, fax, cellphones, applications, and wireless gadgets, telemedicine can aid in the better management of diseases. Reviewing the potential role of telemedicine in the pandemic of infectious diseases with a focus on the Coronavirus disease 2019 (COVID‐19) epidemic was the main goal of this study.

**Methods:**

“Google Scholar,” “PubMed,” “Science Direct,” and “Scopus” databases were searched to collect the papers that identify the advantages and disadvantages of telemedicine in the disease pandemic. Searched keywords include: telepharmacy, telemedicine, remote communication, pandemic(s), epidemic, distant care, distant communication, phone consulation, video conference communication and patient education.

**Results:**

Information and communication technology are crucial, especially when dealing with pandemics of infectious diseases like COVID‐19. Less “in‐person” patient visits to hospitals as a result of telemedicine eventually means less labor for the medical staff, less viral exposure for patients, and ultimately less disease spread. By establishing a bidirectional reciprocal relationship between patients and healthcare providers although they are in separate geographical areas, it can improve patient health status.

**Conclusion:**

Governments are currently facing a significant budgetary burden because to the COVID‐19 pandemic. Since patients are not sent to medical facilities in person, which could be a source of infection, telemedicine reduces disease spread while saving money.

## 1 INTRODUCTION

Coronavirus disease 2019 (COVID‐19) is an infectious disease that causes severe acute respiratory syndrome coronavirus 2 and it has rapidly spread across the globe.^1^, ^2^

This pandemic has had very unfavorable effects on health systems around the world and according to infection rates, its mortality and increased workload on the medical staff, the health system needs to adopt proper methods to prevent and treat this infection and manage underlying diseases and other illnesses.^3^, ^4^, ^5^

Due to their benefits including low cost, speed, information records, and extended communication coverage, information and communication technologies have been focusing a lot of healthcare providers' and policymakers' trend toward offering remote health care services in the past few years.^6^, ^7^ Consumers can now access global health‐related information and services via the internet, which is especially useful during pandemics.^8^, ^9^

**Figure 1:**
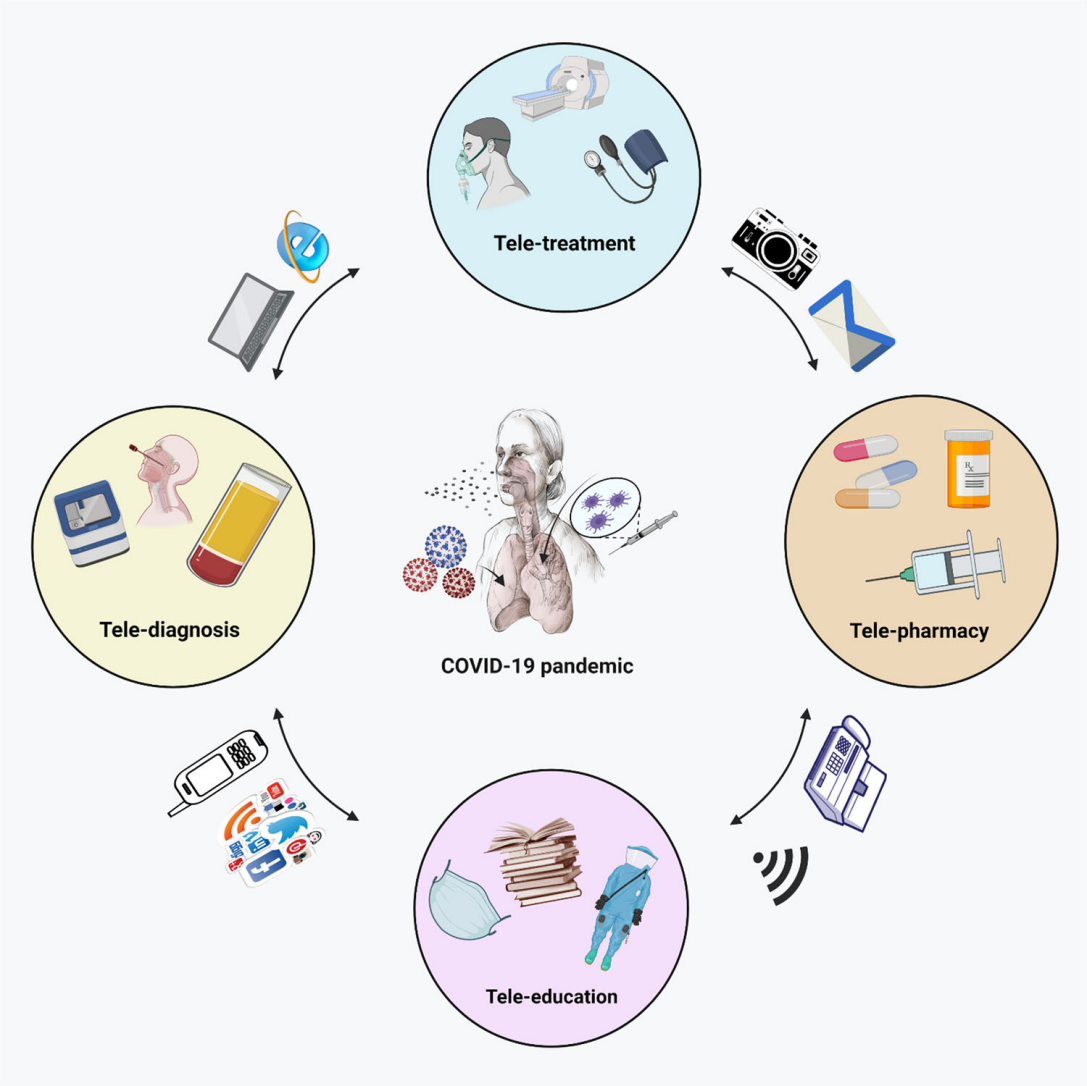
Telemedicine can help to manage diseases better by giving medical or sanitary services to a patient living in other locations via communicational systems such as e‐mail, fax, smartphones, applications, and wireless tools

By creating a bilateral and reciprocal link between patients and care givers even when they are in separate places, telemedicine aims to improve patient health (Figure 1).^10^, ^11^

Telemedicine can evaluate and manage patient conditions, break down distance barriers, and make services more accessible to people in remote places or in situations where patient‐physician contact is difficult.^12^, ^13^

These things can be crucial, especially when dealing with infectious disease pandemics like COVID‐19 because not being familiar with medical facilities can prevent disease spread. Additionally, when telemedicine is employed, information regarding the signs and symptoms, underlying illnesses, and medications taken by patients is recorded in the systems and is accessible to other medical professionals in other locations via communication networks like the internet. Additionally, telemedicine users can share their opinions, strategies, and treatments with their peers who are utilizing the same platform and thereby engaging in the diagnosis, care, and tracking of the patient's development. One branch of telemedicine, for instance, is cogent to distance emergency treatment. Through the use of communicational tools and equipment, doctors working in the central emergency room can consult and direct the first‐responders working in various locations during this procedure.^14^, ^15^

These can be crucial in managing infectious diseases like COVID‐19 since telemedicine allows for better management of the epidemic by employing the expertise of multiple specialists and treating patients in numerous remote medical facilities.^16^

Other responsibilities of telemedicine include fostering contacts and communication between various medical specializations and other members of the care team, such as pharmacists, nurses, and other subject matter experts.^17^

Interdisciplinary interactions are frequently required for patient diagnosis and care to provide the finest and most effective care. This objective can be achieved by telemedicine, which enables communication between a numbers of important professionals despite their geographic separation. This problem can aid in improved illness management, especially when the healthcare system is dealing with an infectious disease pandemic.^18^

In today's health systems, telemedicine is used in a variety of settings and has had positive results in terms of lower costs, fewer hospital admissions, lower patient mortality, and higher patient quality of life. The process of long‐distance practice, which results in such eligible effects, depends on variables like an increase in consult rates along with simplification and improvement of communication between patients and medical professionals.^19^

In a meta‐analysis performed by Gao et al., the use of telemedicine during the COVID‐19 era was evaluated. After deleting duplicates, 2041 articles were discovered in total. Nine studies were ultimately included after reading the complete texts. The results demonstrated that during the COVID‐19 pandemic, people were mainly concerned about the symptoms (64.2%), epidemic condition and public difficulties (14.5%), and psychological problems (10.3%). The percentages of patients seeking consultation for symptoms, preventive and therapy, and psychological issues during the SARS pandemic were 35.0%, 22.0%, and 23.0%, respectively. Telemedicine can be used to screen potential patients and provide guidance, according to two studies. According to one study, it was difficult to track down everyone who calls hotlines and there are few opportunities to identify all suspect situations. They showed that the public's top concerns should be addressed by telemedicine services, which should also give patients with symptoms or those with an epidemic history sensible counsel. These concerns should include symptoms, illness prevention, and disease treatment.^20^

In a clinical trial study that examined the effects of long‐distance medical care on the recovery of 65 patients who underwent knee replacement surgery, it was found that, over the course of a 6‐week course, therapies delivered via long‐distance communication had effects similar to those of therapeutic measures carried out during face‐to‐face visits in terms of rehabilitation outcome. Additionally, patients receiving these services reported higher levels of satisfaction and a higher functional recovery rate after getting patient follow‐up during the rehabilitation phase.^21^

Fourty‐six individuals who donated and received organs in New York were the subjects of a retrospective study. The patients received remote care through frequent phone consultations. Twenty‐four patients had their medications modified throughout the execution of this approach, and six of them required prescription adjustments, according to researchers. As a result, it was discovered in this study that remote medical practice can effectively improve the delivery of healthcare services by boosting interactions between patients and medical personnel.^22^

In a different study conducted in Spain, 83 people with chronic human immunodeficiency virus (HIV) infections were polled. In this study, patients were randomly assigned to two groups: a group of 42 subjects who received routine and standard therapy by hospital in‐person visits over the course of a year, and a group of 41 patients who received the same care over a videoconference. According to the study's findings, 85% of the patients whose treatment and follow‐up were handled through remote medical care said that this approach made it easier and more convenient for them to receive clinical data. They also mentioned how this approach made them feel more at ease.^23^

Studies have revealed that telemedicine‐based psychotherapy has been linked to desired outcomes in a variety of areas, demonstrating that distant medical practice is not restricted to the symptomatic treatment and medication modification of physical ailments.^24^

The psychological toll that the fear of contracting an infectious disease like COVID‐19 and the negative impacts of house quarantine can have on the entire community during a pandemic can be significant. By using this technique, we can treat and even prevent patients from contracting COVID‐19 without their having to come in person.^25^

Bitar et al. carried out a systematic review on patients with chronic diseases and the use of telemedicine during the COVID‐19 pandemic. Overall, 25 of the 51 articles was found matched the requirements for inclusion. To provide healthcare services remotely for people with chronic diseases or conditions in the COVID‐19 era, they consolidated the key findings into ten usages and eight recommendations. They come to the conclusion that there is not much information about these solutions' efficacy.^26^

## 2 METHODOLOGY

In this review we used different search engines such as “Google Scholar,” “PubMed,” “Science Direct,” and “Scopus” for investigating the current articles published on this subject with the aim of finding articles that prove remote communication can be useful especially in patients with infectious diseases, for monitoring patients after surgery, and management of patient care and medication. Some of the main searched keywords include: telepharmacy, telemedicine, remote communication, pandemic(s), epidemic, distant care, distant communication, phone consolation, video conference communication, and patient education.

The cross‐sectional, prospective, and retrospective studies which reported the advantages and disadvantages of telemedicine in the infectious disease pandemic were included for further study. We excluded articles without sufficient data, as well as duplicate publication of same studies, congress abstracts without full texts, and the studies published in other than English language.

## 3 TELEMEDICINE ADVANTAGES

### 3.1 Increasing interprofessional coordination and interaction

Today, multilateral proficient collaboration across many fields is required for disease detection, monitoring, and therapy. Numerous studies show that the presence of a treatment team made up of experts in multiple medical fields improves treatment outcomes when compared to a situation where just one doctor or specialist must diagnose, treat, and manage the disease.^27^, ^28^

By enhancing and streamlining contacts between various specialists and healthcare professionals, telemedicine can contribute to this goal since the treatment of the majority of diseases requires interprofessional cooperation and consensus.^29^

Nine topics about pharmacotherapy in pediatric malignancies were delivered via videoconference sessions in a survey conducted in 2012 to examine the outcome of providing worldwide education via telepharmacy. A total of 345 participants from 10 different nations took part digitally in this investigation. The majority of participants reported that attending these virtual training sessions via telemedicine was associated with positive outcomes, including better practice (*n* = 93), improved patient care (*n* = 82), improved treatment results (*n* = 31), provision for educational needs (*n* = 78), and stabilization and improvement of current practice (*n* = 87).^30^

Researchers observed that the usage of telepharmacy led to an increase in diverse unit personnel interactions and ultimately enhanced employee satisfaction in medical staff, notably nurses, in one of the studies conducted in 2018 to discuss the influence of telemedicine in critical care units. Additionally, it was noted that the implementation of telepharmacy in intensive care unit led to better outcomes for patients' medical concerns.^31^

Higher pharmacologist involvement through telepharmacy can lead to increased accuracy and authenticity of prescriptions and a decrease in the frequency of drug errors because of the significance of the intervention and monitoring of many professionals during the course of drug prescription. On the other hand, telemedicine is the ideal method for treating and managing illnesses when the presence of patients or professionals is not possible, like during the COVID‐19 viral pandemic.^32^, ^33^

### 3.2 Increased data registration and documentation

In telemedicine, information on the medications that doctors or technicians have prescribed is input using a scanner and stored on an in‐network memory. Pharmacologists and other knowledgeable people can then access the recorded data online. From this point forward, pharmacists will be able to review and edit drug prescriptions and match them to patient data and circumstances. The final order is prepared and transmitted to the doctor or technician stationed in another center after pharmacologists make revisions and interventions as directed. As a result, patient data and drug prescriptions are registered, reserved, and documented in the system. Ultimately, this documentation can be used for a patient in a variety of situations.^34^

Researchers found that using telepharmacy increased the rate of documentation and registries of patients' medication data by medical staff, including nurses, clinical pharmacists, and doctors, in a survey to assess the effect of telepharmacy on the correction of medication errors in patient discharge prescriptions.^8^

A review article by Sarkar et al. revealed that there has been a notable increase in the rate of medical therapy recording.^12^

Increased data collection and documentation can improve accuracy by streamlining access to the patient's medical records when needed and facilitating future follow‐up and check‐ups. Additionally, with more data recording, it is easier to track down and find potential mistakes in prescriptions or the distribution of drugs. Probable complications are faster identified and resolving them is easier.^35^

In most cases, data documentation will lead to good disease management, especially in individuals with chronic diseases. Eventually, we will be able to manage patients holistically during times of crisis and infectious disease pandemics when medical staff is under a lot of strain.^36^, ^37^

### 3.3 Lowering medical and pharmacological errors

According to the FDA, a pharmacological error is any avoidable mistake that results in improper drug usage or patient damage while the medication is being administered and being monitored by a professional, patient, or consumer.^38^

A significant portion of the health system's funding is preserved and prevented from being wasted as a result of the codification and management of a strategy to lessen these problems. Medication errors can result in patients receiving medical care suffering irreversible repercussions in addition to financial losses.^8^

By fostering close communication between patients and other medical experts like pharmacologists, telemedicine can therefore play a significant role in reducing medical errors in health systems. This is especially true when medical staff is under a lot of strain during infectious disease pandemics like COVID‐19, which can be the cause of medical errors. In conclusion, telemedicine can communicate between various specialists and consult documents to avoid and remedy medical mistakes in patients.^39^

In some facilities, prescription medications are reviewed retroactively in terms of potential consequences every 30 days. The lengthy duration of this approach, which may have irreversible effects in people taking high‐risk medications, is one of its drawbacks. Additionally, the 30‐day drug recheck method is prone to mistakes and is not helpful for individuals who require more frequent monitoring due to an underlying condition or medications they are taking.^13^

According to a study from Washington, the use of telepharmacy results in the possibility of monitoring drug prescriptions and the elimination of medical mistakes made by pharmacists at night or while they are not in the office.^40^

Therefore, telepharmacy can be used to make up for a medical center's lack of qualified staff members so that patients who are referred to these facilities can receive the same level of medical care as those who are referred to larger facilities.^41^

One hundred patients who were released from the hospital with prescriptions filled participated in a case‐control study. Pharmacologists in this study called patients one week after they were discharged to remind them to take their prescribed medications, assessed how well patients were following their prescriptions, and looked for any mistakes or potential side effects. In this study, researchers found that 61% of patients' discharge summary notes contained inaccuracies, and roughly 75% of patients were at risk from significant clinical concerns. Additionally, compared to the control group, individuals receiving telepharmacy had fewer referrals to acute care units within 30 days after discharge.^42^

Therefore, telemedicine helps reduce medication prescription errors and eliminate patient referral, especially in light of the COVID‐19 outbreak. Eventually, this matter decreases medical staff's workload in medical centers and prevents these patient's exposure to the virus.^43^

### 3.4 Time thrift

According to studies, the technician only inputs medical instructions and prescription data into the system once before sending it to the center in rural areas with access to telemedicine. The time required to enter prescriptions for each patient into the system is reduced because posted data are retained and recorded in the central database. Therefore, technicians working in rural centers and other centers will have more free time to speak with patients and monitor their treatment progress.^44^, ^45^, ^46^

Additionally, telepharmacy makes it feasible for applicants and pharmacologists to communicate over great distances, which reduces the amount of time that patients must spend traveling to cities and larger facilities for consultations and prescription services.^47^, ^48^

Receiving appropriate, high‐quality care close to home without having to move around results in saving time and money for patients, doctors, and healthcare providers. Eventually, during the COVID‐19 pandemic, fewer people will seek out medical facilities, which will lessen the workload of the medical staff and lower the risk of patients contracting the virus.^49^

### 3.5 Simplifying service access

The issue in rural, underdeveloped, or isolated communities is the disparity in services provided compared to larger cities with better‐equipped, more modern, and larger medical facilities. In addition to being of lesser quality, fewer services are provided in villages, and obtaining them presents a number of difficulties.^50^

When the healthcare system is dealing with infectious disease pandemics like COVID‐19,^49^, ^50^, ^51^ telemedicine offers applicants the chance to easily receive their necessary medications and treatment with the same quality and quantity given in larger cities and states regardless of their geographical location of residence.^51^, ^52^, ^53^

Therefore, using telemedicine effectively can help simplify and expand the delivery of medical care to rural, underserved, and remote places, resolving the equipment and human resource shortages in these areas.^54^

### 3.6 Patient consulting improvement

By improving and streamlining professional and patient contacts and communication, distant medical practices can acquire the trust and happiness of their patients. Additionally, it simplifies the process for patients to report any complications. Therefore, face‐to‐face communication using telemedicine increases patient comprehension and awareness and increases their compliance with therapy.^13^

In light of this, telemedicine can increase patient and medical staff communication and lead to patient satisfaction.^55^

A study conducted in the United States investigated the impact of holding training sessions for patients through videoconference on their adherence to prescriptions and proper drug usage. In this study, the intervention group included 77 individuals with a total of 117 pharmacological diets. Following the issuance of prescriptions, medication consultation was carried out for these patients during a training session conducted via videoconference. The control group consisted of 1465 patients with a total of 1736 pharmacological diets who only got medication prescriptions based on the severity of their ailment and who did not participate in a videoconference consult session. The rate of prescription compliance significantly increased in the intervention group that participated in medication consultation via videoconference, according to researchers.^56^

As a result, telemedicine can be utilized to set up multimedia communication to provide instructions on how to administer various medications. Due to this issue, patient knowledge is raised, medication is used appropriately, used drugs are more effective, and in‐person patient referrals to medical facilities are avoided.^56^

Asthmatic residents of rural areas were educated about the disease for three months through sessions held via telepharmacy systems, according to a 2017 survey looking at the effect of telepharmacy on asthmatic patients' access to medical care. Researchers saw that more than 90% of educated patients reached a correctly controlled asthma phase following virtual instruction using telepharmacy, both during the training sessions and in subsequent follow‐ups. As a result, this research suggests that providing asthmatic patients with the necessary training via telepharmacy can help them feel better and be able to regulate their condition.^57^

Researchers examined the impact of telepharmacy on the frequency of inhaler usage by chronic obstructive pulmonary disease (COPD) patients in a random one‐sided blinded clinical trial survey conducted in 2013 in the United states.

Patients with a history of COPD over the age of 60 who used inhalers less frequently than was anticipated were the focus of this investigation. Fourty‐eight patients were included in the control group and 49 were placed in the intervention group. Pharmacologists called these patients in the fourth and eighth weeks following each consultation for a total of 6 months to consult with them and to follow up on their use of inhalers. The findings indicate that patients' use of inhalers has increased as a result of the telepharmacy application. Additionally, all intervention group participants who completed telepharmaceutical consultation said that they felt more satisfied after phone consults for medicine than they did after receiving direct in‐person recommendations. Due to the absence of necessity for in‐person visits and the fact that these patient groups are considered to be at high risk for COVID‐19 infection, the use of telemedicine for COPD control and management is of considerably higher importance.^58^

### 3.7 Expense thrift

Numerous studies have shown that using telepharmacy can encourage thrift and minimize prices for each patient, and doing so results in the reduction of various costs and expenses.^59^, ^60^, ^61^

Frugality in spending and money management are crucial, especially in developing nations where medication costs 25%–66% of the budget for the healthcare system, compared to 10% in rich nations.^30^

When medications are prescribed, dispensed, or used incorrectly, patients, and customers may experience more unpleasant side effects, mortality, and morbidity. Services to address negative drug side effects brought on by medical mistakes cost patients and healthcare systems a lot of money every year. Telemedicine, especially in times of crisis and infectious disease pandemics that place a significant financial burden on health systems, can stop this loss of money and save it by reducing the occurrence of errors when prescribing and dispensing the drugs as well as by quickly identifying potential errors and side effects and solving them.^13^, ^62^, ^63^

### 3.8 Challenges of remote communication

Although telemedicine is an advantageous form of communicating with patients and care givers during difficult times, it is not without challenges. One of the main objections is the presence of a care giver and patient friendly subsystem for communication. These systems, although present, are not without cost. Another disadvantage is the need to educate staff and patients for proper communication and state the importance of knowing when to use such systems. It is vital that patients know the difference between the conditions that need physical contact with a care center and when it is safe to rely on remote communication Table 1.

**Table 1:** A summary of the advantages and challenges for telemedicine in COVID‐19 pandemic

Year of publication	Advantages	Authors	References
2022	COVID‐19 pandemic like wars and natural disasters have resulted in widespread adoption of tele‐critical care.	Ganapathy et al.	64
2020	Direct‐to‐consumer telemedicine can enable patients to connect with their healthcare provider at a distance.	Moazzami et al.	65
	Telemedicine could use of webcam‐enabled computers and smartphones and allows medical practitioner to remarkably screen patients with early signs of COVID‐19 before they reach to hospital.		
2020	Telemedicine connects the low cost, convenience, and ready accessibility of health‐related information and communication using the Internet and associated technologies.	Vidal‐Alaball et al.	66
	Telemedicine could lead to a significant decline in unnecessary patients visit and encouraging self‐quarantine.		
2020	Telemedicine can provide appropriate access to routine care without the risk of exposure in a congested health center waiting rooms.	Smith et al.	67
2020	Tele‐intensive care unit nursing minimized the risk to bedside nurses while maintaining a high level of care for patients.	Arneson et al.	68
Challenges			
2022	Tele‐critical care needs transparent communication with software, hardware, and connectivity as three important components.	Ganapathy et al.	64
	Barriers in implementing tele‐critical care solutions include compensation structures, regulatory policies, cybersecurity, network infrastructure costs, and vulnerabilities.		
	More studies on Tele‐Critical implementation and outcomes is required to develop best practice guidelines, certification, standardization of processes, and clinical training paradigms.		
2022	Because of the COVID‐19 pandemic, it is crucial to develop and update the guidelines and regulations for telemedicine reimbursement.	Salmanizadeh et al.	69
	Future studies can examine the telemedicine reimbursement methods in developed and developing countries before and after the COVID‐19 pandemic.		

## 4 CONCLUSION

The use of telemedicine in a variety of contexts, such as providing medical care to remote areas during emergencies and infectious disease pandemics like the COVID‐19 virus and establishing interactions between specialists and patients, is linked to favorable outcomes like increased interprofessional interactions, improved patient‐staff communication, decreased medication errors, time and cost savings for the health system, and reduced consumable costs. In general, telemedicine can be a useful tool for maintaining public health through interdisciplinary collaboration. When infectious disease pandemics are present, this method can help patients with chronic illnesses avoid face‐to‐face patient consultations, thereby reducing the risk of them contracting the infectious disease and, ultimately, improving the overall state of public health. In conclusion, the COVID‐19 outbreak has put a significant financial strain on governments, and using telemedicine not only saves money but also has the potential to reduce the spread of disease since there are no in‐person referrals of patients to hospitals that could serve as a source of infection.

## AUTHOR CONTRIBUTIONS

**Fazlollah Shokri**: Methodology. **Sara Bahrainian**: Investigation. **Fatemeh Tajik**: Methodology. **Elaheh Rezvani**: Investigation. **Aref Shariati**: Writing – review and editing. **Shima nourigheimasi**: Writing – review and editing. **Elahe Saberi Shahrebabaki**: Writing – original draft. **Maryam Ebrahimi**: Writing – original draft. **Mohsen Heidary**: Conceptualization.

## CONFLICT OF INTEREST

The authors declare no conflict of interest.

## TRANSPARENCY STATEMENT

The lead author Mohsen Heidary affirms that this manuscript is an honest, accurate, and transparent account of the study being reported; that no important aspects of the study have been omitted; and that any discrepancies from the study as planned (and, if relevant, registered) have been explained.

## Data Availability

The authors confirm that the data supporting the findings of this study are available within the article.
